# Preparation and Photocatalytic Performance of In_2_O_3_/Bi_2_WO_6_ Type II Heterojunction Composite Materials

**DOI:** 10.3390/molecules29204911

**Published:** 2024-10-17

**Authors:** Xiuping Zhang, Fengqiu Qin, Yuanyuan Zhong, Tian Xiao, Qiang Yu, Xiaodong Zhu, Wei Feng, Zhiyong Qi

**Affiliations:** 1 School of Mechanical Engineering, Chengdu University, Chengdu 610106, China; zhangxiuping@stu.cdu.edu.cn (X.Z.); mysumeiren@163.com (F.Q.); zhongyuanyuan@stu.cdu.edu.cn (Y.Z.); 13880590027@163.com (T.X.); yuqiang9651@163.com (Q.Y.); 2 Sichuan Province Engineering Technology Research Center of Powder Metallurgy, Chengdu 610106, China; 3 Material Corrosion and Protection Key Laboratory of Sichuan Province, Zigong 643002, China; 4 Institute of Urban Agriculture, Chinese Academy of Agricultural Sciences, Chengdu 610000, China; qizhiyong@caas.cn

**Keywords:** Bi_2_WO_6_, In_2_O_3_, II-type heterojunction, photocatalytic activity

## Abstract

Bismuth-based photocatalytic materials have been widely used in the field of photocatalysis in recent years due to their unique layered structure. However, single bismuth-based photocatalytic materials are greatly limited in their photocatalytic performance due to their poor response to visible light and easy recombination of photogenerated charges. At present, constructing semiconductor heterojunctions is an effective modification method that improves quantum efficiency by promoting the separation of photogenerated electrons and holes. In this study, the successful preparation of an In_2_O_3_/Bi_2_WO_6_ (In_2_O_3_/BWO) II-type semiconductor heterojunction composite material was achieved. XRD characterization was performed to conduct a phase analysis of the samples, SEM and TEM characterization for a morphology analysis of the samples, and DRS and XPS testing for optical property and elemental valence state analyses of the samples. In the II-type semiconductor junction system, photogenerated electrons (*e*^−^) on the In_2_O_3_ conduction band (CB) migrate to the BWO CB, while holes (*h*^+^) on the BWO valence band (VB) transfer to the In_2_O_3_ VB, promoting the separation of photoinduced charges, raising the quantum efficiency. When the molar ratio of In_2_O_3_/BWO is 2:6, the photocatalytic degradation degree of rhodamine B (RhB) is 59.4% (44.0% for BWO) after 60 min illumination, showing the best photocatalytic activity. After four cycles, the degradation degree of the sample was 54.3%, which is 91.4% of that of the first photocatalytic degradation experiment, indicating that the sample has good reusability. The XRD results of 2:6 In_2_O_3_/BWO before and after the cyclic experiments show that the positions and intensities of its diffraction peaks did not change significantly, indicating excellent structural stability. The active species experiment results imply that *h^+^* is the primary species. Additionally, this study proposes a mechanism for the separation, migration, and photocatalysis of photoinduced charges in II-type semiconductor junctions.

## 1. Introduction

The mineralization of the organic pollutants in wastewater using photocatalysis is currently a research hotspot [1,2,3]. Traditional photocatalysts, such as TiO_2_, are mostly wide-bandgap semiconductors that can only generate photogenerated carriers under the action of ultraviolet light, with a limited range of light absorption [4,5,6]. Therefore, finding visible-light-responsive photocatalysts is of great significance. Bi_2_WO_6_-based photocatalytic materials have attracted widespread attention from researchers due to their unique layered structure, good visible light response performance, and relatively narrow bandgap width [7,8]. Zhang et al. [7] prepared Co_3_O_4_ QDs/Bi_2_WO_6_ heterojunction photocatalysts using hydrothermal and ultrasonic synthesis and evaluated the photocatalytic performance of the composite material with tetracycline (TC) as the target pollutant. The results of the photodegradation experiment showed that the pseudo-first-order kinetic constant for the degradation of TC by 10%-Co_3_O_4_ QDs/Bi_2_WO_6_ was 0.017 min^–1^, which was 3.40 and 1.55 times higher than that of Co_3_O_4_ QDs and Bi_2_WO_6_. Yang et al. [8] utilized a simple one-step hydrothermal method to prepare oxygen-deficient F-doped Bi_2_WO_6_ (F-Bi_2_WO_6_). Compared to pristine Bi_2_WO_6_, F-Bi_2_WO_6_ exhibited a superior performance in photocatalytic activity, photogenerated charge carrier separation, and visible light absorption. On this basis, the optimized F_1.00_-Bi_2_WO_6_ photocatalytic degradation of MO increased by 2.5 times compared to that of the original Bi_2_WO_6_. However, the quantum utilization efficiency of pure Bi_2_WO_6_ is low, leading to a decrease in its photocatalytic activity. Numerous studies have shown that constructing semiconductor heterojunctions by coupling semiconductors can improve the transfer efficiency of photoinduced charges at the interface, reduce recombination, and thereby enhance photocatalytic activity [9,10,11]. Kuang et al. [10] prepared BiOIO_3_/BiOBr semiconductor heterojunction composite materials using a hydrothermal method. After 80 min of illumination, the degradation rate of TC could reach 74.91%, which is higher than pure BiOBr (48.12%) and pure BiOIO_3_ (52.24%). Maraj et al. [11] synthesized CdO/Ag_3_PO_4_ composite materials. Under light conditions, due to the band difference between CdO and Ag_3_PO_4_, photogenerated electrons on the CB of CdO will transfer to the CB of Ag_3_PO_4_, and photogenerated holes on the VB of Ag_3_PO_4_ will transfer to the VB of CdO, promoting the separation of photoinduced carriers and improving quantum utilization efficiency.

On the other hand, In_2_O_3_ possesses advantages such as good chemical stability, non-toxicity, harmlessness, and a strong photoresponse ability and is widely used in the field of photocatalysis [12,13,14]. Sun et al. [13] constructed a novel In_2_O_3_/BiFeO_3_ heterojunction by modifying nanoparticles on the surface of BiFeO_3_ nanosheets. Under simulated sunlight irradiation, the degradation of TC showed that the 10.7% IO/BFO composite material had the best photocatalytic activity, which was 2.05 times and 3.97 times higher than In_2_O_3_ and BiFeO_3_. Khaokhajorn et al. [14] synthesized In_2_O_3_/ZnO nanocomposites by the co-precipitation method and used rhodamine B as the target pollutant for photocatalytic degradation to evaluate its photocatalytic performance. The degradation degree of 2% In_2_O_3_/ZnO is 55.5% after 330 min of illumination. Through a series of characterization analyses and experiments on active species, the mechanism of the photocatalytic degradation of rhodamine B was investigated. It was found that a Z-type charge transfer mechanism was formed when ZnO and In_2_O_3_ were coupled, which inhibited the recombination of photogenerated electron–hole pairs in ZnO and In_2_O_3_ and enhanced the photocatalytic degradation efficiency of rhodamine B.

Here, we are building upon our previous work, where we successfully synthesized Bi_2_WO_6_ photocatalysts [15]. In order to introduce semiconductor coupling with BWO to form a semiconductor junction, accelerate the transfer of photogenerated charges, and improve quantum efficiency and photocatalytic activity, in this work, In_2_O_3_/Bi_2_WO_6_ type II semiconductor composite photocatalytic materials were prepared using a multi-step synthesis method. The samples were analyzed for their phase composition, morphology, chemical state, and specific surface area by XRD, SEM, TEM, XPS, and BET. Based on the DRS test results, the band potentials of the samples were determined. The generation, migration, and separation of photogenerated charges were analyzed based on PL spectra and electrochemical test results. The separation, migration, and photocatalytic mechanism of the photogenerated charges in In_2_O_3_/Bi_2_WO_6_ type II semiconductor composite photocatalytic materials were proposed based on the above characterizations and experimental results.

## 2. Results

### 2.1. Phase Composition

**Figure 1:**
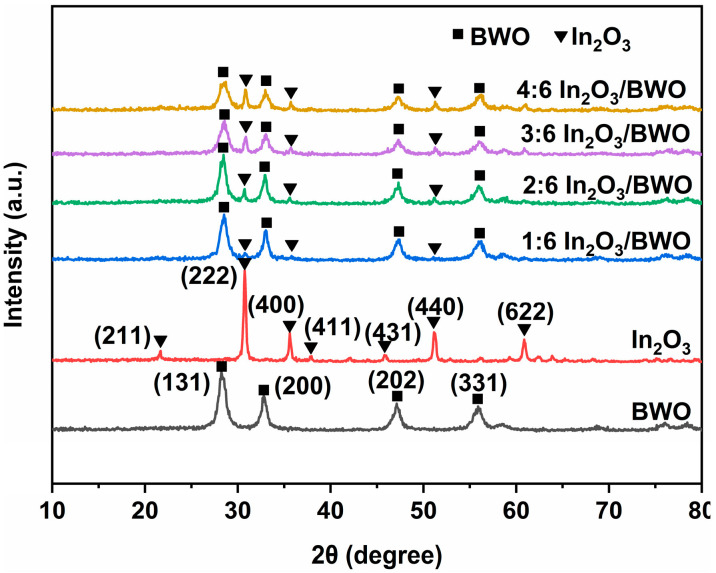
XRD patterns of samples.

The XRD patterns are exhibited in Figure 1. The diffraction peaks of the pattern (BWO) at 28.3°, 32.8°, 47.2°, and 55.8° are attributed to the (131), (200), (202), and (331) planes of BWO, respectively [15,16,17,18]. The diffraction peaks of the pattern (In_2_O_3_) around 21.7°, 30.7°, 35.6°, 37.8°, 45.9°, 51.1°, and 60.9° are attributed to the (211), (222), (400), (411), (431), (440), and (622) planes of In_2_O_3_ [19,20]. After coupling with In_2_O_3_, the main peak positions still correspond to BWO, and, with the increase in the molar ratio of In_2_O_3_/BWO, the peak intensity of In_2_O_3_ in the composite material gradually increases, indicating that In_2_O_3_/BWO composite materials were formed.

### 2.2. Morphology

**Figure 2:**
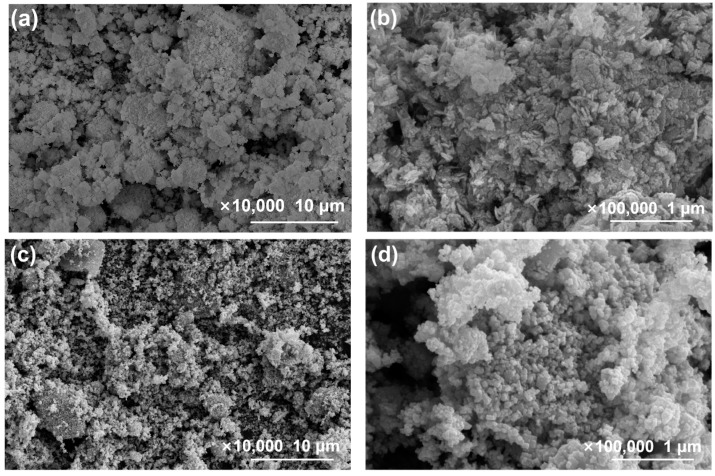

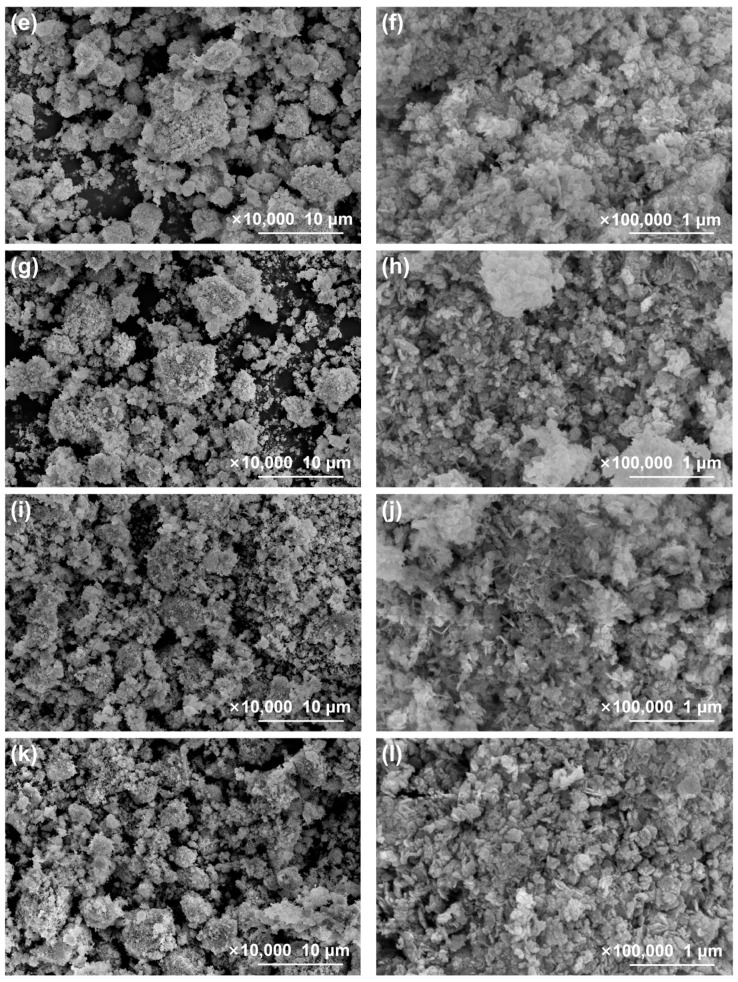
SEM images of samples, with different magnifications: BWO (**a**,**b**), In_2_O_3_ (**c**,**d**), 1:6 In_2_O_3_/BWO (**e**,**f**), 2:6 In_2_O_3_/BWO (**g**,**h**), 3:6 In_2_O_3_/BWO (**i**,**j**), and 4:6 In_2_O_3_/BWO (**k**,**l**).

Figure 2 shows the SEM images of the samples. From Figure 2a,b, the morphology of pure BWO appears to be irregular nanosheets. In Figure 2c,d, In_2_O_3_ is observed to be particles of varying sizes with slight aggregation. In the In_2_O_3_/BWO composite material, the aggregation of In_2_O_3_ is reduced, and it is dispersed on the surface of the BWO nanosheets.

**Figure 3:**
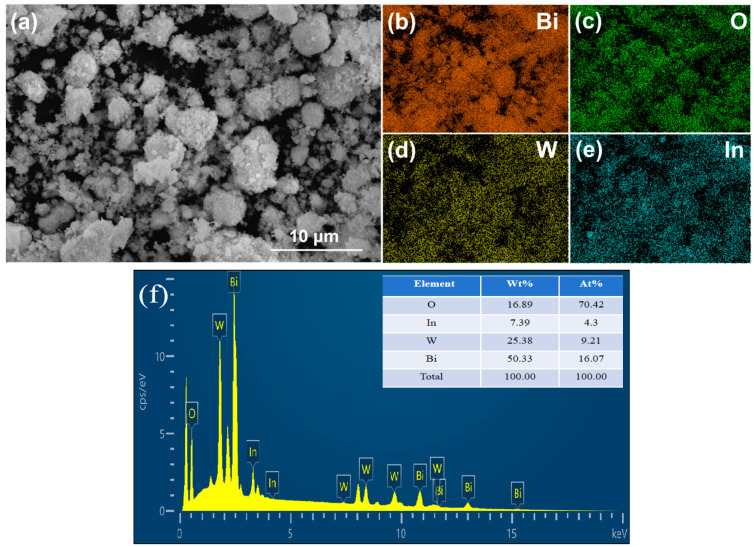
(**a**) SEM image of 2:6 In_2_O_3_/BWO; (**b**–**e**) element mappings of Bi, O, W, In; (**f**) EDS analysis of 2:6 In_2_O_3_/BWO.

The EDS mappings of 2:6 In_2_O_3_/BWO are shown in Figure 3. The mapping shows that the 2:6 In_2_O_3_/BWO photocatalytic material contains four elements, Bi, O, W, and In, which are uniformly dispersed in the composite photocatalytic material, and their atomic percentages are shown in Figure 3f.

**Figure 4:**
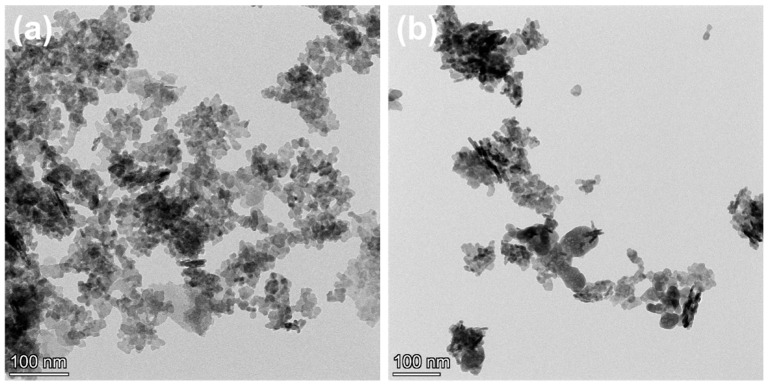

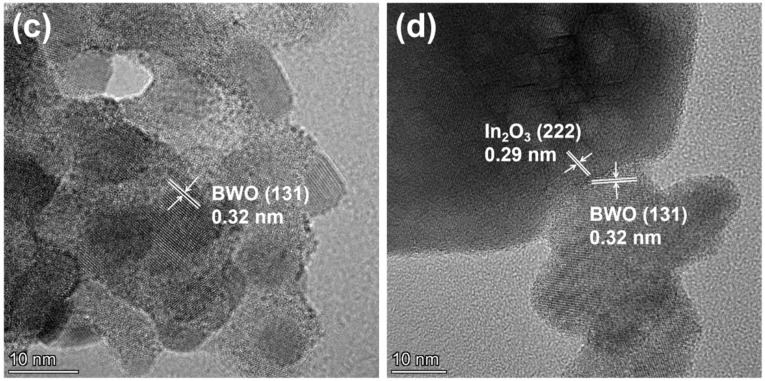
TEM and HRTEM images of samples: BWO (**a**,**c**) and 2:6 In_2_O_3_/BWO (**b**,**d**).

Combining the images in Figure 2 and Figure 4a,b, it can be observed that In_2_O_3_ nanoparticles are distributed on the BWO nanosheets. The crystal plane spacing marked in Figure 4c (0.32 nm) corresponds to the (131) crystal plane of BWO [7,21]. The crystal plane spacings marked in Figure 4d (0.32 nm and 0.29 nm) are ascribed to the BWO (131) and In_2_O_3_ (222) planes [19,22].

### 2.3. Chemical State and Surface Area

**Figure 5:**
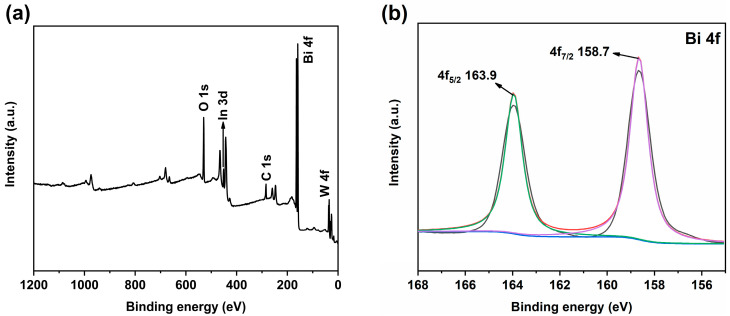

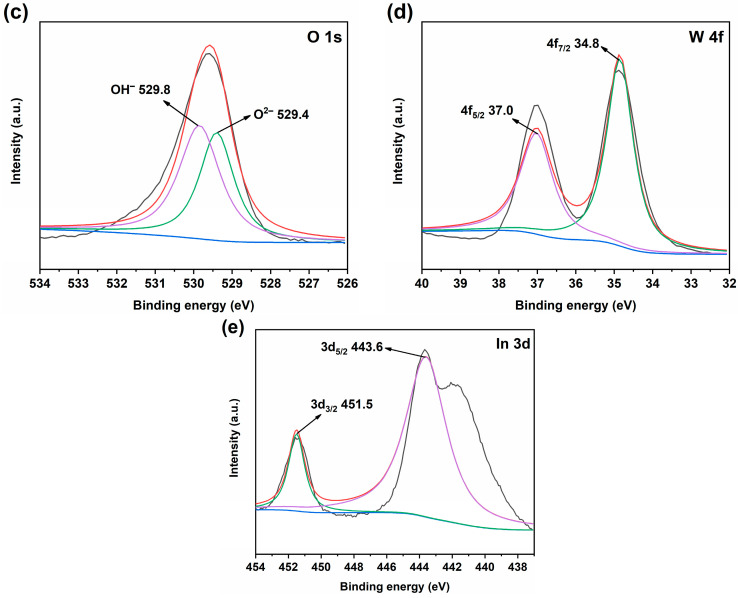
XPS spectra of 2:6 In_2_O_3_/BWO: (**a**) total spectrum; (**b**) Bi 4f; (**c**) O 1s; (**d**) W 4f; and (**e**) In 3d.

The surface element composition and chemical valence state of 2:6 In_2_O_3_/BWO were analyzed by XPS testing. The XPS spectra of 2:6 In_2_O_3_/BWO are presented in Figure 5. The full XPS spectrum of 2:6 In_2_O_3_/BWO reveals signals corresponding to Bi 4f, O 1s, W 4f, and In 3d, demonstrating the existence of these four elements in 2:6 In_2_O_3_/BWO, consistent with the EDS mappings in Figure 3. In the high-resolution Bi 4f spectrum, the characteristic peaks appearing around 158.7 eV and 163.9 eV are ascribed to Bi 4f_7/2_ and Bi 4f_5/2_, revealing the existence of Bi as a +3 valence (Figure 5b) [16,23]. The peaks at 529.4 eV and 529.8 eV in Figure 5c are indexed to lattice oxygen and surface hydroxyl groups [5]. The high-resolution W 4f spectrum in Figure 5d displays characteristic peaks at around 34.8 eV and 37.0 eV, representing W 4f_7/2_ and W 4f_5/2_ and revealing the presence of W as a +6 valence [24,25]. The peaks in Figure 5e at 443.6 eV and 451.5 eV correspond to In 3d_5/2_ and In 3d_3/2_, indicating the existence of In as a +3 valence [12,26,27].

The specific surface area of photocatalysts has a significant impact on their performance. Different research results indicate that after the formation of composite photocatalysts, their specific surface area may increase or decrease. There are reports in the literature that increasing the specific surface area leads to better photocatalytic performance. Wang et al. [12] constructed mesoporous In_2_O_3_/In_2_S_3_ heterojunction photocatalytic materials by in situ generation. By changing the amount of thioacetamide used, the content of In_2_S_3_ can be adjusted, and the specific surface area of In_2_O_3_/In_2_S_3_-2 can reach 129 m^2^/g, which is significantly increased compared with that of In_2_O_3_ (90.1 m^2^/g). Using rhodamine B and phenol as target pollutants, the photocatalytic activity of In_2_O_3_/In_2_S_3_ heterojunctions was studied. The results showed that the degradation kinetic constants of In_2_O_3_/In_2_S_3_-2 for rhodamine B and phenol were 0.0468 and 0.0312 min^−1^, which were 5 times and 3 times that of In_2_O_3_, respectively. On the contrary, some studies suggest that a decrease in specific surface area leads to better photocatalytic performance. Chen et al. [3] constructed La_2_Ti_2_O_7_/Ag_3_PO_4_ photocatalysts using an in situ precipitation method. BET tests showed that the specific surface area of La_2_Ti_2_O_7_/Ag_3_PO_4_ photocatalysts was lower than that of pure La_2_Ti_2_O_7_. The photocatalytic experiment results showed that after 90 min of illumination, the degradation rate of rhodamine B by La_2_Ti_2_O_7_ alone was 11.31%, while, after the same reaction time, the degradation rates of La_2_Ti_2_O_7_/Ag_3_PO_4_-1 and La_2_Ti_2_O_7_/Ag_3_PO_4_-5 were 99.07% and 100%.

**Figure 6:**
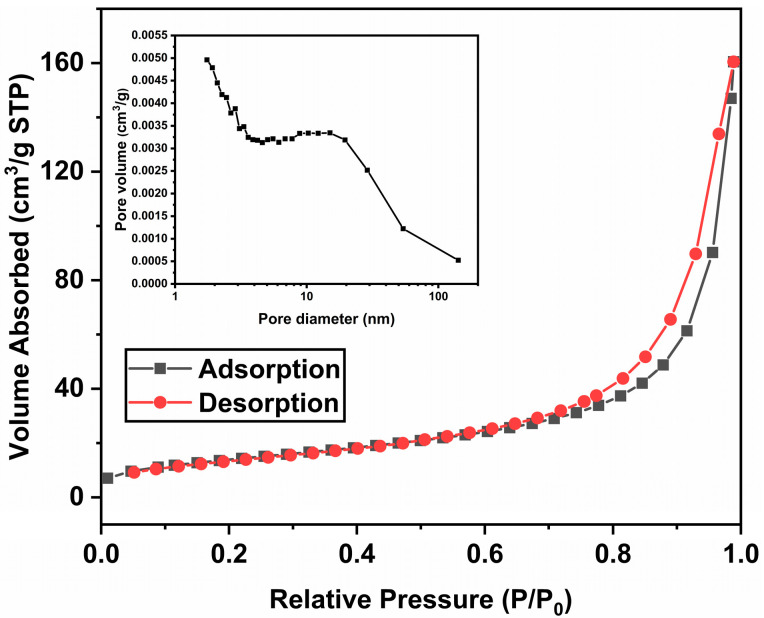
N_2_ adsorption–desorption isotherms and pore size distribution curve of 2:6 In_2_O_3_/BWO.

In this study, the specific surface area of 2:6 In_2_O_3_/BWO was increased, and, combined with subsequent photocatalytic degradation experiments, it was found that its photocatalytic activity was enhanced. Figure 6 shows the N_2_ adsorption–desorption isotherm of 2:6 In_2_O_3_/BWO. It can be observed that the adsorption–desorption isotherm belongs to type IV, and an H3-type hysteresis loop is evident in the relative pressure range of 0.8 to 1, indicating the presence of mesoporous structures in the sample [28,29,30,31]. In previous work, we have measured the specific surface area of pure Bi_2_WO_6_. Compared with pure Bi_2_WO_6_ (50.7 m^2^/g), the specific surface area of the 2:6 In_2_O_3_/Bi_2_WO_6_ (51.0 m^2^/g) photocatalytic material is slightly increased [15]. There are more reaction sites available for the photocatalytic reaction owing to the incremental specific surface area contributing to the enhancement of photocatalytic activity [32,33]. The pore size distribution model of BJH was used to calculate the pore size distribution, and the BJH average pore size of pure Bi_2_WO_6_ and 2:6 In_2_O_3_/BWO was 20.5 nm and 18.7 nm, respectively [15]. Smaller pore sizes can increase light scattering and reflection, thus increasing the interaction between the light source and the sample and improving its photocatalytic activity.

### 2.4. Optical Property

**Figure 7:**
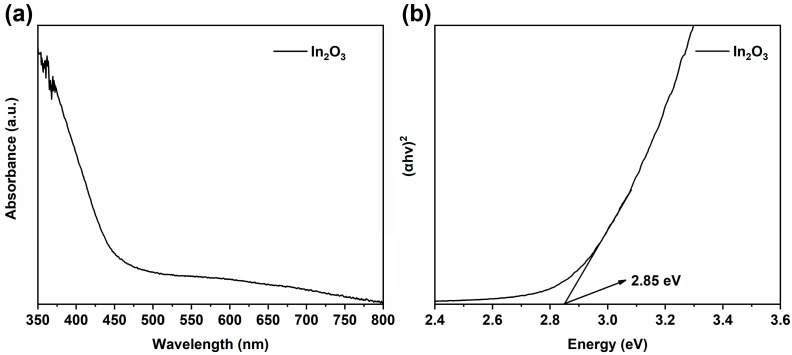
UV–visible diffuse reflectance spectrum (**a**) and bandgap diagram (**b**) of In_2_O_3_.

The UV–visible diffuse reflectance spectrum of In_2_O_3_ is shown in Figure 7a. The results indicate that In_2_O_3_ responds in the UV–visible region. The bandgap (Eg) of semiconductor photocatalysts can be determined using the Kubelka–Munk formula [15,27]:

αhν = A (hν − Eg)^1/n^ (1)

Here, α, h, v, and A represent the optical absorption coefficient, Planck constant, photon frequency, and constant, respectively. The value of n is related to the type of semiconductor. BWO is a direct bandgap semiconductor with n = 1/2, and the bandgap width of BWO is 2.43 eV [15]. In this study, In_2_O_3_ is an indirect bandgap semiconductor n = 2 [27], and the bandgap width of In_2_O_3_ can be obtained from the relationship between (αhν)^1/2^ and hν, as shown in Figure 7b. The bandgap of In_2_O_3_ is 2.85 eV.

**Figure 8:**
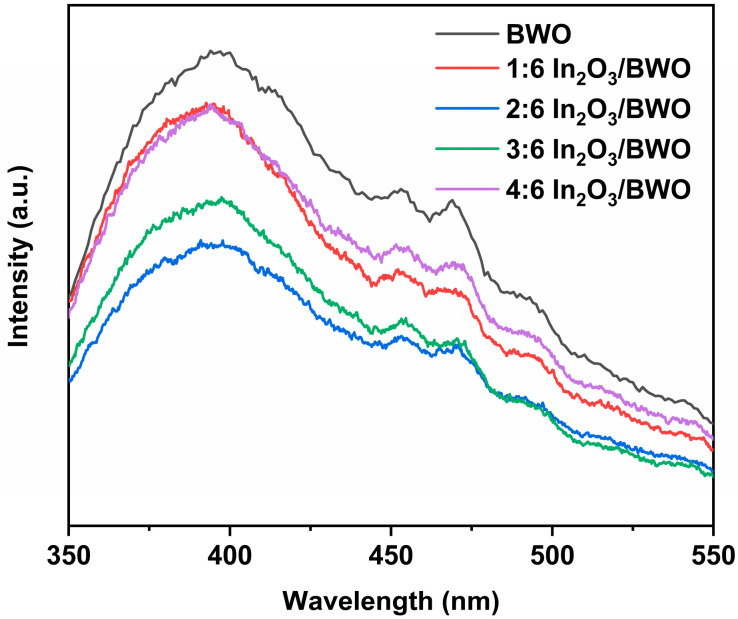
PL patterns of samples.

Photoluminescence (PL) spectra were collected to reflect the recombination of photoinduced charges during the photocatalytic process, with the results shown in Figure 8. Generally, the weaker the PL peak intensity, the lower the probability of a photogenerated electron returning to the VB and binding with its hole, suggesting that more photogenerated charges have the opportunity to play a role in the photocatalytic process [34,35]. The PL peak intensities of In_2_O_3_/BWO composite materials are lower than that of BWO [15], indicating that coupling In_2_O_3_ with BWO helps to suppress the recombination of photogenerated charge carriers. With the increase in the molar ratio of In_2_O_3_/BWO, the PL peak intensity first decreases and then increases. The 2:6 In_2_O_3_/BWO composite material exhibits the lowest PL peak intensity, while the 4:6 In_2_O_3_/BWO composite material shows a higher PL peak intensity. At lower molar ratios of In_2_O_3_/BWO, the different band structures of In_2_O_3_ and BWO facilitate the separation and transfer of photoinduced charges, inhibiting their recombination. However, at higher molar ratios, the formation of new recombination centers may lead to an increase in PL peak intensity [36].

**Figure 9:**
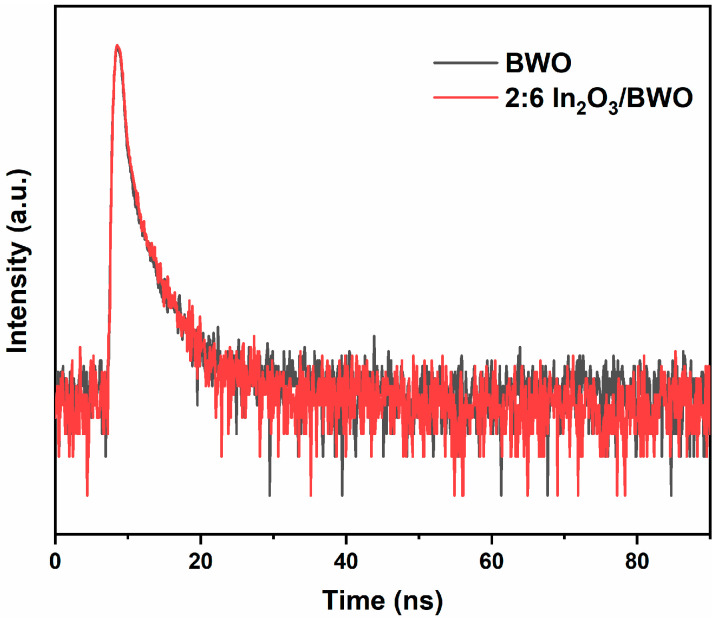
Time-resolved transient PL decay of BWO and 2:6 In_2_O_3_/BWO.

To further verify the separation of photogenerated charges, the time-resolved fluorescence spectra of the BWO and 2:6 In_2_O_3_/BWO photocatalytic materials were tested, and the results are shown in Figure 9. The fluorescence decay curves were fitted according to the principle of the double exponential kinetic function, as shown below [3,21]:

I(t) = B_1_exp (−t/τ_1_) + B_2_exp (−t/τ_2_) (2)

where B_1_ and B_2_ represent the amplitude and τ_1_ and τ_2_ represent the corresponding emission lifetime. In addition, in order to evaluate the recombination of photogenerated carriers, the average lifetime (τ_ave_) can be determined by the following formula. The calculation results of B_1_, B_2_, τ_1_, τ_2_, and τ_ave_ are summarized in Table 1.

τ_ave_ = (B_1_τ_1_^2^ + B_2_τ_2_^2^)/(B_1_τ_1_ + B_2_τ_2_) (3)

A large number of studies have shown that the longer the fluorescence lifetime, the greater the probability of a photogenerated charge migrating to the surface of the sample, thus generating more free radicals, which is conducive to improving photocatalytic efficiency [37,38,39,40]. Chen et al. [37] synthesized a Ag_3_PO_4_/P-g-C_3_N_4_ photocatalytic material by a microwave-assisted heating and ion exchange two-step method and found that the longer the fluorescence lifetime, the higher the photogenerated charge separation efficiency, and the better the photocatalytic performance of the composite photocatalytic material. Zografaki et al. [38] prepared CeO_2_/g-C_3_N_4_ (CN) composites with different concentrations by a simple wet chemical solution method. Time-resolved fluorescence spectroscopy was used to study the photoinduced carrier dynamics and determine the fluorescence lifetime of CN and 10% CeO_2_/CN composites. The results show that the fluorescence lifetime of the 10% CeO_2_/CN composite is relatively longer than that of pure CN, which may be due to the synergistic effect and close interface contact between CN and CeO_2_, resulting in a decrease in the photogenerated electron and hole recombination rate and an enhancement of photogenerated carrier separation, which is conducive to improving quantum efficiency and photocatalytic activity. In this study, the fluorescence lifetime of In_2_O_3_ combined with Bi_2_WO_6_ was extended from 0.82 ns to 0.85 ns. Combined with the results of subsequent photocatalytic degradation experiments, it can be seen that this study is consistent with reports in the literature [37,38,39,40].

### 2.5. Photodegradation Results

**Figure 10:**
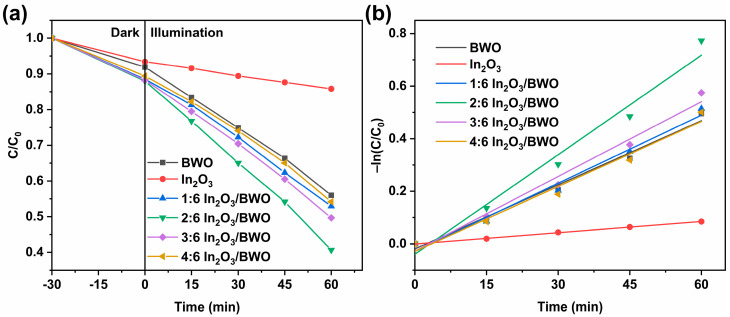
Photodegradation curves (**a**) and kinetic fitting curves (**b**) of samples.

Figure 10a presents the photodegradation curves of the samples. After 60 min of illumination, the results for 1:6 In_2_O_3_/BWO, 2:6 In_2_O_3_/BWO, 3:6 In_2_O_3_/BWO, and 4:6 In_2_O_3_/BWO are 47.1%, 59.4%, 50.0%, and 45.8%, respectively; all higher than pure BWO (44.0%) and pure In_2_O_3_ (14.2%) [15]. When the molar ratio of In_2_O_3_/BWO is 2:6, it exhibits the most optimal photocatalytic activity of all samples. This may be attributed to the formation of a type II semiconductor structure when In_2_O_3_ is coupled with BWO, inhibiting the reunion of photoinduced charges and enhancing its photodegradation activity.

Figure 10b shows the kinetic fitting curves of the samples. In kinetic studies, photocatalytic degradation follows the pseudo-first-order kinetic model, whose expression is as follows [13,41,42]:

−ln(C/C_0_) = k_app_t (4)

where C and C_0_ represent the initial concentration of rhodamine B and the concentration of rhodamine B at time t during the degradation process, respectively; k_app_ is a pseudo-first-order kinetic constant; and t represents the corresponding degradation time. The pseudo-first-order kinetic constant (k_app_) for BWO, In_2_O_3_, 1:6 In_2_O_3_/BWO, 2:6 In_2_O_3_/BWO, 3:6 In_2_O_3_/BWO, and 4:6 In_2_O_3_/BWO is 0.008 min^−1^, 0.001 min^−1^, 0.009 min^−1^, 0.013 min^−1^, 0.010 min^−1^, and 0.008 min^−1^, respectively [15]. Among them, the k_app_ of 2:6 In_2_O_3_/BWO is 1.6 times that of BWO and 13.0 times that of In_2_O_3_.

**Figure 11:**
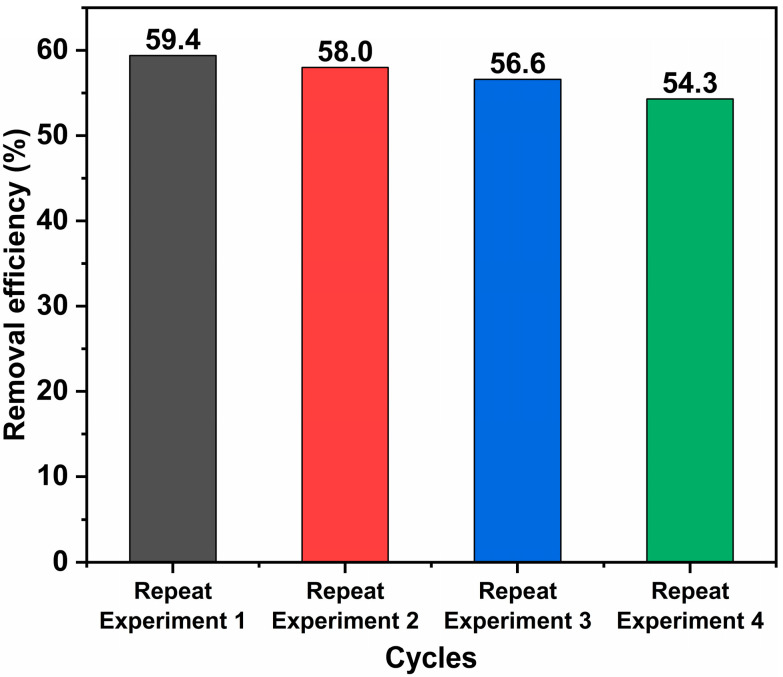
The reuse experiment of 2:6 In_2_O_3_/BWO for RhB degradation.

The cycling experiment results for 2:6 In_2_O_3_/BWO are shown in Figure 11. After four cycles, the degradation of RhB by 2:6 In_2_O_3_/BWO decreases from 59.4% to 54.3%, possibly due to partial sample loss during the sample recovery process.

**Figure 12:**
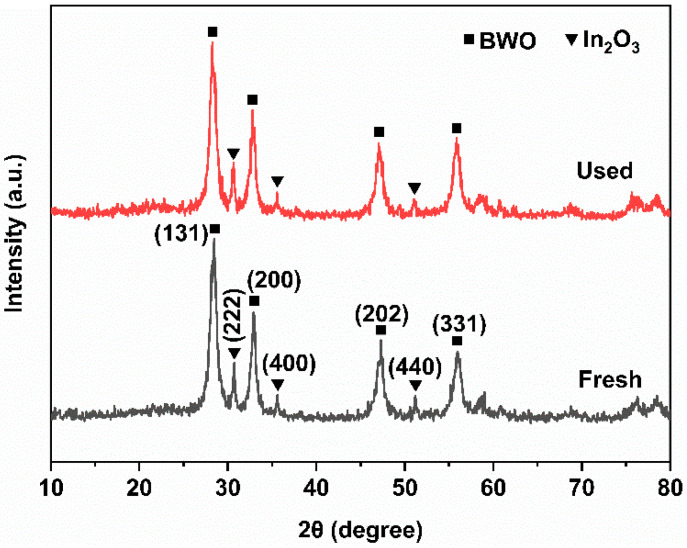
XRD patterns of 2:6 In_2_O_3_/BWO before and after the photocatalytic experiment.

Figure 12 shows the XRD patterns of the fresh and used 2:6 In_2_O_3_/BWO samples. The intensity and position of the XRD diffraction peaks of the sample almost remain unchanged before and after the photocatalytic experiments, indicating that the prepared 2:6 In_2_O_3_/BWO exhibits good structural stability.

**Figure 13:**
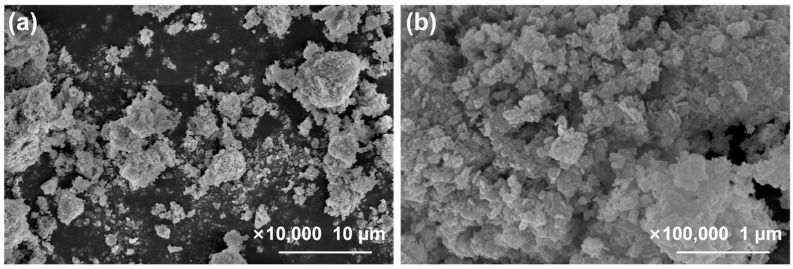
SEM images of 2:6 In_2_O_3_/BWO at 10,000× magnification (**a**) and 100,000× magnification (**b**) after cycling experiments.

Figure 13 shows SEM images of 2:6 In_2_O_3_/BWO after the cycle experiment. When compared with Figure 2g,h, it can be observed that there were no significant changes in the particle morphology of the samples before and after the cycling experiment.

**Figure 14:**
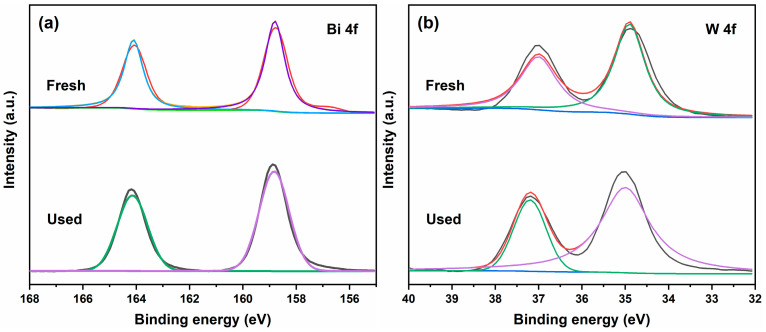

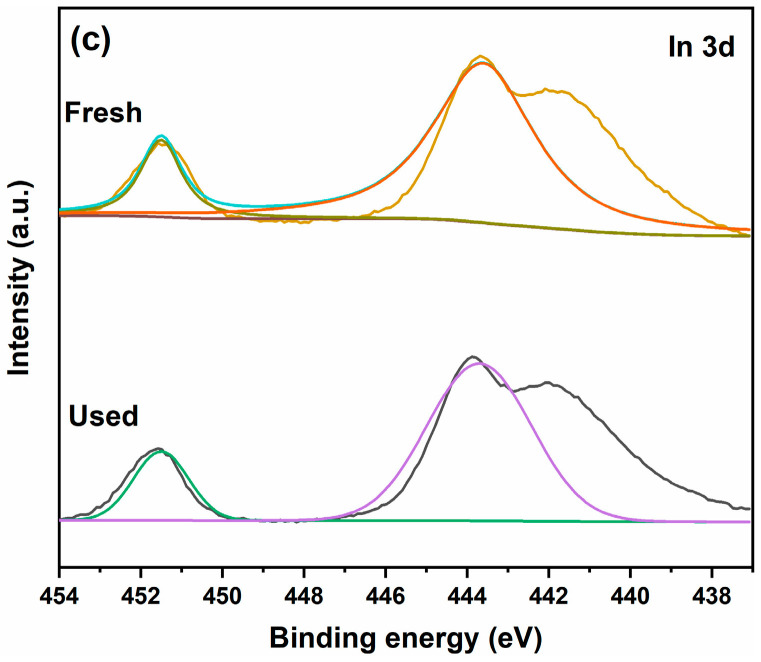
XPS spectra of 2:6 In_2_O_3_/BWO before and after the photocatalytic experiment: (**a**) Bi 4f; (**b**) W 4f; (**c**) In 3d.

Figure 14 shows the XPS pattern of the 2:6 In_2_O_3_/BWO photocatalytic material after the cycling experiment. Compared with the unused sample, the position of its XPS peak did not change after the cycling experiment. It can be seen that the Bi, W, and In elements are still a +3, +6, and +3 valence before and after use, indicating that the chemical valence states have not changed.

### 2.6. Photodegradation Mechanism

**Figure 15:**
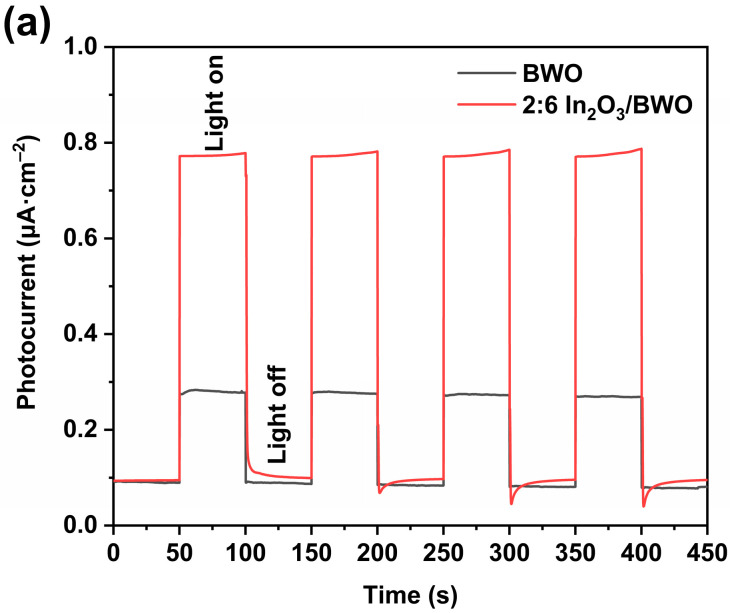

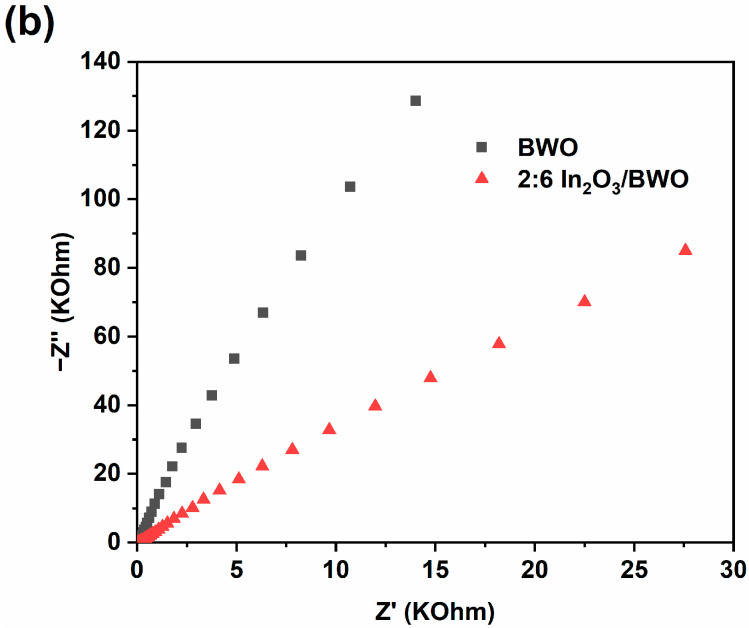
Photocurrent response curves (**a**) and electrochemical impedance spectroscopy curves (**b**) of BWO and 2:6 In_2_O_3_/BWO.

The separation and migration characteristics of the photogenerated charges in semiconductor photocatalytic reactions are crucial factors. To further explore the charge transfer mechanism at work during the semiconductor photocatalytic reaction, the authors introduced electrochemical testing on the basis of time-resolved fluorescence spectra and a PL characterization analysis. When the photon energy received by semiconductor materials is greater than their bandgap width, valence band electrons will transition to the conduction band, generating photogenerated charges. The directional movement of the conduction band electrons and valence band holes will generate a photocurrent. The magnitude of the photocurrent density reflects the strength of the photogenerated charge separation ability. A higher photocurrent density means that the corresponding photocatalyst has a better ability to separate photogenerated electrons and holes [43,44]. The photocurrent response was employed to analyze the separation of photoinduced charges in BWO and 2:6 In_2_O_3_/BWO samples, as shown in Figure 15a. The photocurrent density of 2:6 In_2_O_3_/BWO is much stronger than that of BWO, revealing that the cooperation of In_2_O_3_ promotes the separation of photogenerated charge carriers in the composite material [15]. Electrochemical impedance spectroscopy (EIS) was used to analyze the migration of photogenerated charges in the samples, as shown in Figure 15b. According to the Nyquist theorem, the smaller the radius of the impedance arc, the lower the corresponding resistance to photogenerated charge migration [41,45,46]. The Nyquist arc radius of 2:6 In_2_O_3_/BWO is smaller than that of BWO, indicating that 2:6 In_2_O_3_/BWO has a smaller charge migration resistance, facilitating the migration of photoinduced charges to the catalyst surface for participation in photocatalytic oxidation reactions [15].

**Figure 16:**
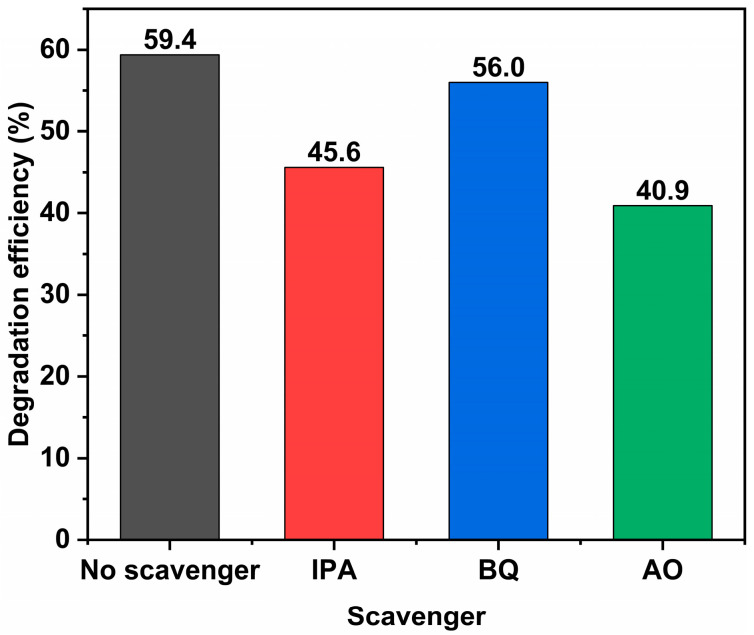
The degradation degrees of 2:6 In_2_O_3_/BWO in the presence of different scavengers.

To investigate the dominant active species responsible for the photocatalytic degradation process of the 2:6 In_2_O_3_/BWO composite material, isopropyl alcohol (IPA), benzoquinone (BQ), and ammonium oxalate (AO) were used as radical scavengers to capture hydroxyl radicals (·OH), superoxide radicals (·O_2_^−^), and holes (*h*^+^) in the sample [5,13]; the results are shown in Figure 16. The experimental results show that the addition of these scavengers reduced the degradation degree of 2:6 In_2_O_3_/BWO from its original 59.4% (without scavengers) to 45.6% with IPA, 56.0% with BQ, and 40.9% with AO. The addition of the AO collector had the greatest effect on the degradation of 2:6 In_2_O_3_/BWO, while the addition of IPA and BQ led to slight decreases in degradation, indicating that *h*^+^ are the primary active species in the photodegradation process of 2:6 In_2_O_3_/BWO; ·OH play a secondary role, and ·O_2_^−^ have a relatively minor impact.

The energy band potentials of In_2_O_3_ are determined using Equations (5) and (6), which are specified as follows [47,48,49]:

E_CB_ = X − E_e_ − 1/2E_g_ (5)

E_VB_ = E_g_ + E_CB_ (6)

E_e_ is about 4.5 eV, and the X value of In_2_O_3_ is 5.28 eV. According to Figure 7b, the bandgap of In_2_O_3_ is 2.85 eV, so the E_CB_ and E_VB_ of In_2_O_3_ are −0.65 eV and 2.20 eV, respectively. In our previous research, the bandgap width of BWO was 2.43 eV, and the E_CB_ and E_VB_ of BWO were calculated to be 0.68 eV and 3.11 eV, respectively [15].

If the photogenerated charge transfer pathway in the 2:6 In_2_O_3_/BWO photocatalyst follows a Z-scheme mechanism, under the condition of illumination, the photogenerated electrons in the Bi_2_WO_6_ conduction band will migrate to the valence band of In_2_O_3_ and compound with the holes in the valence band of In_2_O_3_, thus retaining photogenerated electrons in the In_2_O_3_ conduction band. The electrons in the In_2_O_3_ conduction band can accumulate and participate in the reduction reaction, and a relatively large number of superoxide radicals will be retained in the In_2_O_3_ conduction band. This is inconsistent with the experimental results of the active species in this study. Therefore, the In_2_O_3_/BWO photocatalyst does not form a Z-type heterostructure.

**Figure 17:**
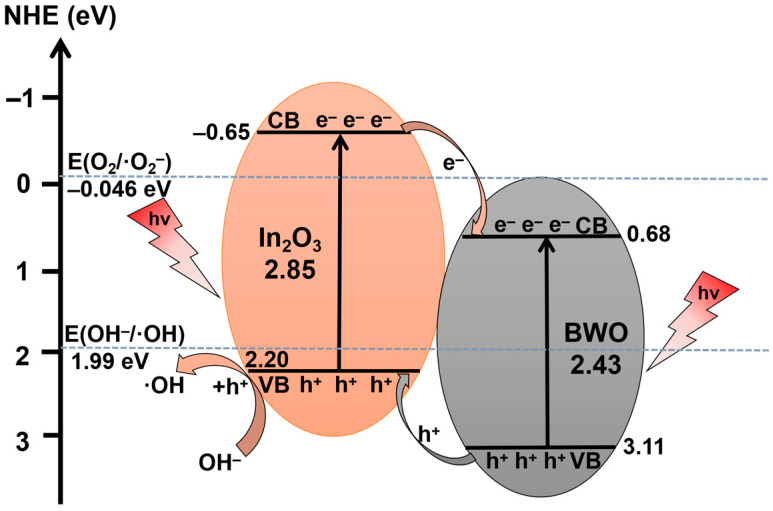
Schematic diagram of photogenerated charge transfer and formation of free radicals in 2:6 In_2_O_3_/BWO.

In previous work, the authors conducted XPS testing on pure Bi_2_WO_6_, where the Bi 4f spectrum of Bi_2_WO_6_ decomposed into two characteristic peaks located at 158.8 eV and 164.1 eV, corresponding to Bi 4f_7/2_ and Bi 4f_5/2_ [15]. In this study, the Bi 4f of the 2:6 In_2_O_3_/BWO composite was decomposed into two characteristic peaks, which appear near 158.7 eV and 163.9 eV, belonging to Bi 4f_7/2_ and Bi 4f_5/2_ and indicating that Bi is +3. Compared to pure Bi_2_WO_6_, the Bi element in the 2:6 In_2_O_3_/BWO composite material exhibits a shift towards a lower binding energy, revealing an electron transfer from In_2_O_3_ to Bi_2_WO_6_ [50,51,52]. Therefore, a type II 2:6 In_2_O_3_/BWO semiconductor junction photogenerated charge separation and transfer mechanism is proposed, as shown in Figure 17. Due to the fact that the CB potential of BWO (0.68 eV) is lower than the CB potential of In_2_O_3_ (−0.65 eV), when BWO is coupled with In_2_O_3_, photogenerated electrons on the In_2_O_3_ CB transfer to the BWO CB. Simultaneously, as the VB potential of BWO (3.11 eV) is also lower than the VB potential of In_2_O_3_ (2.20 eV), photogenerated holes on the BWO VB migrate to the In_2_O_3_ VB. This migration of photoinduced electrons and holes effectively suppress the recombination of photoinduced charges, thereby enhancing quantum efficiency. Additionally, the VB potential of In_2_O_3_ aligns with OH^−^/·OH = 1.99 eV, enabling photogenerated holes on the In_2_O_3_ VB to react with OH^–^, producing ·OH radicals [53]. In the degradation process, the RhB molecule is decomposed into small inorganic molecules such as CO_2_ and H_2_O, with hydroxyl free radicals and holes as the main active groups [54,55].

We have summarized the photocatalytic properties of some photocatalytic materials, as shown in Table 2. Mandal et al. [56] prepared CeO_2_-TiO_2_ nanocomposites by mechanical alloying. The photodegradation of a model organic pollutant rhodamine B has been investigated using a UV-vis spectrophotometer under visible light illumination. About 63% of rhodamine B was degraded under visible light in the presence of the 20CeO_2_-80TiO_2_ nanocomposite within 240 min. By exploring the mechanism of its photocatalysis, the results show that a heterojunction is formed between CeO_2_ and TiO_2_, which promotes the transfer of photogenerated charges between the interface, improves the quantum efficiency, and enhances photocatalytic activity. Feng et al. [45] successfully prepared g-C_3_N_4_/Bi_4_O_5_I_2_ composites by heat treating a g-C_3_N_4_/BiOI precursor at 400 °C for 3 h. The photocatalytic properties of g-C_3_N_4_/Bi_4_O_5_I_2_ photocatalytic materials were evaluated by the photodegradation of MO in visible light. After 40 min of visible light irradiation, 20% g-C_3_N_4_/Bi_4_O_5_I_2_ has the best photocatalytic performance, and its degradation rate reaches 0.164 min^−1^, which is 3.2 times that of Bi_4_O_5_I_2_. According to the band potential and active species experiments of photocatalytic materials, it is proposed that photogenerated electrons and holes move through the type II mechanism, which effectively inhibits the recombination of photogenerated electrons and holes, prolongs the carrier lifetime, and thus enhances photocatalytic activity. The type II heterojunctions formed in this study are similar to those reported in the literature.

## 3. Materials and Methods

### 3.1. Materials

Bi(NO_3_)_3_·5H_2_O, Na_2_WO_4_·2H_2_O, InCl_3_·4H_2_O, glacial acetic acid, ethylene glycol, anhydrous ethanol, isopropyl alcohol (IPA), benzoquinone (BQ) and ammonium oxalate (AO) were procured from Chengdu Kolon Chemical Co., Ltd., Chengdu, China. It is worth noting that all the chemical reagents mentioned above were of analytical grade.

### 3.2. Sample Preparation

Bi(NO_3_)_3_·5H_2_O (4 mmol) and glacial acetic acid (15 mL) were added to ethylene glycol (30 mL) to fully dissolve and obtain solution A. Na_2_WO_4_·2H_2_O (2 mmol) was added to ethylene glycol (20 mL) to obtain solution B. Solution B was added to solution A through a separating funnel. We transferred the obtained mixed solution to a 100 mL reaction kettle and heated it at 180 °C for 10 h. Deionized water and ethanol were used to wash the precipitation alternately; after drying, pure Bi_2_WO_6_ powder was obtained. It was labeled as BWO.

A total of 3 mmol InCl_3_·4H_2_O and 2 mmol NaOH were added to 50 mL of deionized water and stirred for 1 h. The resulting mixed solution was transferred to the inner liner of a reaction vessel and hydrothermally treated at 160 °C for 12 h. After the hydrothermal treatment, the sample was rinsed with anhydrous ethanol and purified water alternately and dried at 80 °C for 10 h, followed by grinding to obtain a white powder. Finally, the powder was calcined at 600 °C and kept at this temperature for 1 h to obtain In_2_O_3_.

During the preparation of Bi_2_WO_6_, a suitable amount of In_2_O_3_ was added. We ensured that the preparation process and conditions were consistent with those of Bi_2_WO_6_, resulting in the synthesis of In_2_O_3_/Bi_2_WO_6_ composite photocatalytic materials with different molar ratios (x = 1:6, 2:6, 3:6, 4:6). They were, respectively, labeled as 1:6 In_2_O_3_/BWO, 2:6 In_2_O_3_/BWO, 3:6 In_2_O_3_/BWO, and 4:6 In_2_O_3_/BWO.

### 3.3. Characterization

An X-ray diffractometer (XRD, DX-2700B) from Dandong Haoyuan Instrument Co. Ltd., located in Dandong, China, with a scan range 2θ of 10°–80°, scan speed of 0.06°/s, voltage of 40 kV, and current of 30 mA, was used to analyze the crystal structure and phase information. The samples’ morphology was assessed through a scanning electron microscope (SEM, FEI-nspect F50) with an operating voltage of 5 kV and a Tecnai G2 F20 transmission electron microscope (TEM and HRTEM) from FEI Company, situated in Hillsboro, OR, USA, with an acceleration voltage of 200 kV. X-ray Photoelectron Spectroscopy (XPS) was performed using an instrument from Thermo Scientific K-Alpha, provided by Kratos Ltd., Manchester, UK; its operating potential is 12 kV and filament current is 6 mA. This was used to analyze the elemental valence state composition. A Brunauer–Emmett–Teller (BET, ASAP 2460) from the Guoyi Precision Measurement Technology Co. Ltd., Beijing, China was measured the pore size and surface area; a UV-Visible Spectrophotometer (DRS, UV-3600) from the Shimadzu Group Company, Kyoto, Japan analyzed the optical absorption; a Fluorescence Spectrophotometer (PL, F-4600) from the Shimadzu Group Company in Kyoto, Japan, with an excitation wavelength of 300 nm and a wavelength range from 350 to 550 nm, was used to detect and analyze the recombination of photoinduced charges; and an Electrochemical Workstation (DH7000) supplied by Jiangsu Donghua Analytical Instrument Co., Ltd., based in Jingjiang, China, evaluated the photocurrent curves (PCs) and electrochemical impedance spectroscopy (EIS) of the samples, analyzing the separation and migration of photogenerated charges. A Pt electrode, Ag/AgCl electrode, and 0.1 mol/L Na_2_SO_4_ were used as the opposite electrode, reference electrode, and electrolyte, respectively.

### 3.4. Photocatalysis Experiment

A solution of RhB dye (10 mg/L) was used to evaluate the samples’ photocatalytic activity. A total of 15 mg of the sample was added to 100 mL of RhB aqueous solution. The homogeneous mixture was stirred for 30 min in the dark, followed by photocatalytic degradation experiments under 250 W xenon lamp irradiation. The absorbance of the solution was tested every 15 min, the solution was centrifuged to collect the upper clear liquid, and the λ was set at 553 nm to test the absorbance of the liquid. The degradation degree (η) can be calculated by the formula

η = [(A_0_ − A_t_)/A_0_] × 100% (7)

where A_0_ and A_t_ are the initial and t time absorbance values.

### 3.5. Active Species Experiments

The main active groups were analyzed by using IPA to capture ·OH, BQ to trap ·O_2_^−^, and AO to scavenge *h*^+^. In the ·OH capture experiment, 2 mL of IPA was added to the photocatalyst and RhB mixture. The capture experiments for ·O_2_^−^ and *h^+^* were conducted similarly to the ·OH experiment. The concentration of all trapping agents was 2 mmol/L.

## 4. Conclusions

In summary, In_2_O_3_/BWO composite materials were prepared through a multi-step synthesis method. SEM and EDS tests reveal that the agglomeration of In_2_O_3_ particles was reduced and they were dispersed on the surface of BWO nanosheets. Particles of In_2_O_3_ were in close contact with the BWO nanosheet, forming a type II semiconductor structure. The photogenerated electrons on the In_2_O_3_ CB transfer to the BWO CB, and the photogenerated holes on the BWO VB transfer to the In_2_O_3_ VB, promoting carrier separation. From the perspective of optical properties and an electrochemical analysis, this structure is conducive to the rapid transfer of photogenerated charges at the contact interface and suppresses the aggregation of photogenerated charges. The highest photocatalytic activity was observed when the atomic ratio of In_2_O_3_ to BWO was 2:6. The degradation degree of RhB was 59.4% after 60 min of irradiation with the 2:6 In_2_O_3_/BWO photocatalyst. The results of the experiments with active species suggest that photogenerated *h^+^* is the predominant active species during the course of photodegradation.

## Figures and Tables

**Table 1:** Exponential decay-fitted parameters of fluorescence lifetime of BWO and 2:6 In_2_O_3_/BWO.

Samples	B_1_	τ_1_ (ns)	B_2_	τ_2_ (ns)	τ_ave_ (ns)
BWO	1546.0089	0.46	263.2821	2.95	0.82
2:6 In_2_O_3_/BWO	1680.5211	0.48	309.1260	2.87	0.85

**Table 2:** The summarization of the pollution degradation degrees of various photocatalysts.

Catalyst	Method	Catalyst (mg/L)	Pollutant (mg/L)	Light Source	Degradation Degree	Ref.
C_3_N_4_-MoS_2_	Hydrothermal	80	6.4/RhB	Sunlight (1000 W/m^2^)	40.3% (30 min)	[57]
CeO_2_-TiO_2_	Ball milling	20	4/RhB	Filament lamp (200 W)	63.0% (240 min)	[56]
In_2_O_3_/ZnO	Co-precipitation	1000	10/RhB	Blacklight blue lamps (18 W)	55.5% (300 min)	[14]
Mo@Ni-MOF	Solvothermal approach	500	10/MB	Solar irradiation	57.4% (60 min)	[58]
NiFe_2_O_4_/rGO	Hydrothermal	20	10/MG	Tungsten lamp (200 W)	38.9% (60 min)	[59]
2:6 In_2_O_3_/Bi_2_WO_6_	Solvothermal approach	150	10/RhB	Xenon lamp (250 W)	59.4% (60 min)	This work

## Data Availability

Data are contained within the article.
